# Antibiotic prescribing among patients with severe infectious diseases in two private sector hospitals in Central India – a time series analysis over 10 years

**DOI:** 10.1186/s12879-020-05059-7

**Published:** 2020-05-13

**Authors:** Anna Damlin, Megha Sharma, Gaetano Marrone, Cecilia Stålsby Lundborg

**Affiliations:** 1 Department of Global Public Health, Health Systems and Policy. Karolinska Institutet, SE-171 77 Stockholm, Sweden; 2 Department of Molecular Medicine and Surgery, Division of Clinical Physiology, Karolinska Institutet, SE-171 76 Stockholm, Sweden; 3 Department of Pharmacology, Ruxmaniben Deepchand Gardi Medical College, 456006 Surasa, Ujjain, India

**Keywords:** Antibiotics, Prescribing practice, Bacterial infections, Antibiotic resistance, Fixed dose combinations, Private-sector hospitals

## Abstract

**Background:**

Antibiotic resistance is an emerging problem caused due to antibiotic use. In countries with high rates of infectious diseases, antibiotic resistance is a frequent cause of mortality. The aim was to analyse antibiotic prescribing practices between 2008 and 2017 in a teaching (TH) and a non-teaching (NTH) hospital, as typical hospitals of low- and middle-income countries, and to compare antibiotic prescribing for severe infectious indications for which empiric antibiotic treatment is recommended.

**Methods:**

Data from adult patients registered at two Indian private-sector hospitals with one of the following indications: epiglottitis, pneumonia, peritonitis, pyelonephritis, cellulitis, erysipelas, septic arthritis, endocarditis, meningitis or sepsis; were included and analysed. Antibiotic prescription data was analyzed using the World Health Organization’s (WHO) Anatomical Therapeutic Chemical classification system and the Defined Daily Doses. Chi-square and linear regression were used to compare the data between groups. Time series analyses were conducted using linear regression. *P*-values < 0.05 were considered significant.

**Results:**

In total, 3766 patients were included, 2504 inpatients in the NTH and 1262 in the TH, of which 92 and 89% patients, respectively, were prescribed antibiotics. Sixty-one percent of total prescriptions in the TH and 40% in the NTH comprised the *access* category of antibiotics (i.e. the first-choice of treatment according to the WHO). The WHO’s second-choice of treatment, the *watch* category, comprised 29 and 40% of total prescriptions in the TH and NTH, respectively. Prescribing of fixed-dose combinations (FDCs) of antibiotics was significantly higher in the NTH (18%) than in the TH (8%, *P* < 0.05). Prescribing of *watch* antibiotics and FDCs increased significantly in both hospitals between 2008 and 2017 among patients with pneumonia, cellulitis and peritonitis (*P* < 0.05).

**Conclusions:**

Prescribing of *watch* antibiotics and FDCs of antibiotics increased over time at both hospitals, indicating under prescribing of *access* antibiotics and more prescribing of second-choice antibiotics. The results can be used to highlight the areas of improvement in similar settings. Implementing diagnostic routines and local prescribing guidelines could improve the prescribing practices.

## Background

Antibiotic resistance is an emerging global threat, as it causes significant morbidity and mortality worldwide [1, 2]. In an antibiotic surveillance report between 2016 and 2017 that focused on 22 countries, bacteria resistant to at least one of the most commonly used antibiotics were identified in patients diagnosed with bloodstream infections [3]. According to the World Health Organization (WHO), there is a need for global action against antibiotic resistance to ensure the effectiveness of antibiotic treatment in the future [4].

In low- and middle-income countries (LMICs), the infectious disease burden is often high, and antibiotic resistance is one of the common causes of mortality among patients with infectious diseases [2, 5]. Tackling antibiotic resistance requires costly equipment for microbiological analyses to determine the susceptibility of bacteria, and these methods must be implemented and reported [5, 6]. In LMICs, access to diagnostic methods is often limited. Consequently, antibiotics, frequently broad-spectrum antibiotics or fixed-dose combinations (FDCs), are commonly prescribed empirically based on a clinical suspicion of infection [7–10]. However, antibiotic resistance is not confined to LMICs.

Improper use of antibiotics contributes to the development of antibiotic resistance. As reported previously, antibiotics should be prescribed only for relevant indications [11]. Furthermore, they should be as targeted as possible and administered at correct doses for appropriate treatment durations and by a suitable route of administration [11]. Mapping of antibiotic prescribing practices can identify areas for improvement. Long-term studies on antibiotic prescribing in LMICs, especially studies comparing practices among various health care providers and in various settings, are scarce [6].

The study population was inpatients admitted to the study hospitals, with severe infections for which empirical antibiotic treatment is recommended [12]. The hospitals selected for this study were representative of the majority of healthcare facilities in LMICs. The primary aim was to present and compare antibiotic prescribing practices over a 10-years period in two private-sector hospitals in Ujjain, Madhya Pradesh, India. A secondary aim was to compare antibiotic prescribing practices for selected indications with global recommendations for antibiotic treatment.

## Methods

### Study settings

This prospective study with time-series analyses was conducted at two tertiary care, private sector hospitals run by the same trust, located in Ujjain district of Madhya Pradesh, India, one teaching hospital (TH) and one non-teaching (NTH). The TH is in a rural area and has 800 beds and the NTH is centrally located with 400 beds. At the TH, patients are provided medical services and medicines free of charge while medical services at the NTH are charged but at a reduced level [13]. At the NTH, the patients purchase their medicines also during hospital stay. Medical representatives are not allowed to visit the prescribers at the TH while they can do so at the NTH. A local essential medicines list was available at the TH, though it was not completely implemented but no local prescribing guidelines were available at the TH or NTH. As none of the hospitals had computerized prescribing records, data was manually registered in a form inserted in each patient’s medical file at time of admission, and prospectively filled during the patients stay in the hospital. This was made by trained nursing staff that completed forms continuously, which has been described in detail earlier [9, 13].

### Categorization of antibiotics

Prescribed antibiotics were classified using the Anatomical Therapeutic Chemical classification system (ATC) and the Defined Daily Doses (DDD) classification (2019) according to the WHO [14]. The WHO has classified antibiotics based on the risk of antibiotic resistance development into so-called *access, watch* and *reserve* antibiotic categories [4, 15–17]. This classification of antibiotics was adopted for analysis (Table 1). The aim for the categorization of antibiotics is to sort antibiotics according to how they should be used, based on the risk of development of antibiotic resistance to preserve the effectiveness of antibiotic treatment and to improve clinical outcomes [4, 15, 16]. *Access* antibiotics should be widely available, affordable and of good quality, *watch* antibiotics include most of the highest priority, critically important medicines and should be used only for specific and limited indications and *reserve* antibiotics should only be used when all alternative antibiotics have been unsuccessful for the treatment [4, 15, 16]. Some of the prescribed antibiotics are categorized only up to antibiotic-groups level, these antibiotics were added to the relevant category as per their antibiotic-groups. For example; cefuroxime was not categorized by the WHO but second generation cephalosporins were categorized as *access* antibiotic, then we added cefuroxime to the *access* category for the analysis [17]. Since FDCs consist of at least two antibiotics, often from different antibiotic-groups, we added “FDCs” as a category beside the *access*, *watch* and *reserve* antibiotics. Antibiotics categorized in the respective categories are presented in Table 1.

**Table 1:** Antibiotics categorized in *access, watch, reserve* and fixed dose combinations of antibiotics

ATC-code	Antibiotic group	Specific antibiotics	Antibiotic category
J01A	Tetracyclines	Doxycycline	*Access*
		Tigecycline	*Reserve*
J01B	Amphenicols	Chloramphenicol	*Access*
J01C group 1: J01CA, J01CE, J01CF, J01CG	Penicillins with extended spectrum, Beta-lactamase sensitive penicillins, beta-lactamase resistant penicillins, beta-lactamase inhibitors	Amoxicillin, ampicillin, benzathine benzylpenicillin, benzylpenicillin, cloxacillin, phenoxymethylpenicillin, procaine benzylpenicillin, piperacillin, tazobactam	*Access*
J01CR	Combinations of penicillins including beta-lactamase inhibitors	Amoxicillin with clavulanic acid	*Access*
		Piperacillin with tazobactam	*Watch*
J01D	Beta-lactam antibiotics	Cefalexin, cefazolin, cefadroxile, cefradine, cefuroxime	*Access*
		Cefixime, ceftriaxone, cefotaxime, cefoperazone, cefodoxime, ceftazidime, meropenem, imipenem, cilastin, faropenem	*Watch*
		Aztreonam, cefepime, ceftaroline	*Reserve*
J01E	Sulfonamides and trimethoprim	Sulfamethoxazole with Trimethoprim	*Access*
J01F	Macrolides	Clindamycin	*Access*
		Azithromycin, clarithromycin, erythromycin, lincomycin, roxithromycin	*Watch*
J01G	Aminoglycosids	Gentamicin, netilmicin, kanamycin, tobramycin, streptomycin, amikacin	*Access*
J01M	Quinolones and fluoroquinolones	Ciprofloxacin, levofloxacin, moxifloxacin, norfloxacin, orfloxacin, gemifloxacin, pazufloxacin, gatifloxacin, prulifloxacin	*Watch*
J01R	Combinations of antibiotics	Ampicillin with Cloxacillin. Amoxicillin with Cloxacillin Azithromycin with Ambroxol Cefixime with Ornidazole Cefoperazone with Sulbactam Ceftriaxone with Sulbactam Ceftriaxone with Tazobactam Norfloxacin with Tinidazole Ofloxacin with Ornidazole Ofloxacin with Tinidazole Cefixime with Clavulanate Potassium Cefixime with Clavulanic Acid Cefixime with Cloxacilline Cefixime with Ofloxacin Cefixime with Tazobactam Cefotaxime with Sulbactam Cefpodoxime with Clavulanic Acid Cefpodoxime with Cloxacillin Cefpodoxime with Dicloxacillin Meropenem with Sulbactam Ceftazidime with Tazobactam Cefuroxime with Clavulanic Acid Ciprofloxacin with Ornidazole Ciprofloxacin with Tinidazole Efoperazone with Sulbactam Levofloxacin with Ornidazole Cefixime with Azithromycin Cefpodoxime with Potassium Clavulanate Ceftriaxone with Clavulanic Acid	FDCs of antibiotics
J01X	Other antibiotics	Metronidazole (J01XD01), nitrofurantoin, tinidazole, ornidazole, spectinomycin	*Access* (P01AB01 Metronidazole included)
		Teicoplanin, vancomycin	*Watch*
		Polymyxin B, colistin, fosfomycin, linezolid, daptomycin	*Reserve*

*Abbreviations*: *ATC* Anatomical Therapeutic Chemical Classification, *FDC* Fixed dose combination

### Data analysis

Antibiotic prescribing data was collected prospectively from the records of all patients, admitted to the TH and the NTH between April 1st 2008 and May 22nd 2017. The study population comprised inpatients with severe infections for which empiric antibiotic treatment was indicated by the WHO [15]. Data from all adult patients (≥18 years) that stayed at least one night in either hospital, and diagnosed with any of the following infectious indications- epiglottitis, pneumonia, peritonitis, pyelonephritis, cellulitis, erysipelas, septic arthritis, infective endocarditis, meningitis and sepsis were screened for the analyses. However, fewer patients were registered with epiglottitis, pyelonephritis, erysipelas, septic arthritis, infective endocarditis and meningitis, therefore, data from the inpatients with pneumonia, peritonitis, cellulitis and sepsis were selected for detailed analysis. A unique code was generated for each patient record, without identifying the patients individually, thus all data were anonymized. Patient data were analyzed for gender, duration of hospital stay and if antibiotics were prescribed or not during hospital stay. The antibiotic prescription data were analyzed for type of antibiotic, dose, treatment duration, frequency and route of administration. To analyze the adherence to prescribing guidelines, existing international guidelines for empiric antibiotic prescribing were used [15, 18, 19].

Prescribed antibiotics were grouped for their first 4–5 characters of their ATC-code: J01A, J01B, J01C group 1 (containing all antibiotics starting with J01CA to J01CG), J01CR, J01D, J01E, J01F, J01G, J01M, J01R and J01X (Table 1) [14]. The J01R contained the FDCs of antibiotics that has been listed in the ATC/DDD classification system until June 2019. Prescribed antibiotics were also classified for: *access, watch, reserve* and FDCs of antibiotics (Table 1) [4, 15, 16]. Antibiotic prescribing was calculated for in DDDs and DDD per 1000 patient days according to following formulas:

$$ DDD\kern0.5em per\kern0.5em prescription\kern0.5em =\kern0.5em \frac{dose\kern0.5em in\kern0.5em grams\kern0.5em \times \kern0.5em frequency}{WHO\kern0.5em DDD\kern0.5em for\kern0.5em the\kern0.5em prescribed\kern0.5em antibiotic} $$

$$ DDD\kern0.5em per\kern0.5em 1000\kern0.5em patient\kern0.5em days\kern0.5em =\frac{DDD_{total}\kern0.5em \ast \kern0.5em 1000/ 365}{N} $$

Where DDD_total_ is total antibiotic prescribing (in DDDs) prescribed during one year among a patient group and N is total number of patients in that patient group during that year*.*Time series analyses were conducted using linear regression for antibiotic prescribing with DDDs per 1000 patient days as dependent variable and year as independent variable to obtain a slope for the trend over the study period. For categorical variables, frequencies and percentage were calculated. For numerical variables, sum and mean with its 95% confidence interval (CI) were calculated. Chi-square test (for categorical variables) and linear regression (for continuous numerical variables) were used to compare the data between the two hospitals and between the patient groups. Pearson chi-square was used for expected values > 5 and Fischer’s exact test for expected values < 5. *P*-values < 0.05 were considered statistically significant. Data was entered manually in EPI Info 3.1 and analyzed using STATA software version 15.1 (Stata Corp. College Station. Texas. USA).

## Results

**Fig. 1:**
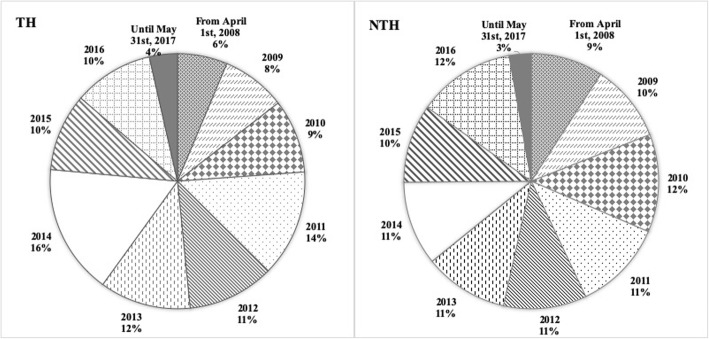
Distribution of the included patients at each hospital at each year of the study period. Legend. Distribution of included patients is presented in percentage of patients included each year out of all included patients, at each hospital. Abbreviations: NTH, non-teaching hospital; TH, teaching hospital

In total, 134,666 patients were admitted to the NTH, and 109,108 patients were admitted to the TH between 2008 and 2017. Data from 3766 patients were included in the analysis (NTH: *n* = 2504; TH: *n* = 1262, Table 2, Fig. 1). Overall, the patients from the TH were younger than the patients from the NTH (mean age NTH: 49.2, TH: 47.1 years, *P* < 0.01). At both hospitals, there were smaller proportions of women admitted, compared to men (percentage of admitted women NTH: 36%, TH: 24%) (Table 3). Antibiotics were commonly prescribed in both hospitals, although a significantly higher percentage of patients admitted to the NTH were prescribed antibiotics as compared with those admitted to the TH (89% at the TH, 92% at the NTH, *P* < 0.05) (Table 3). Among the different diagnostic groups, there were no differences in antibiotic prescribing practices between the hospitals, except for meningitis, where 90% of the patients in the NTH were prescribed antibiotics as compared with 70% in the TH (*P* < 0.05). The number of antibiotic prescriptions per patient in the TH was higher than that in the NTH. The average number of antibiotic prescriptions, i.e. prescription of one specified antibiotic with stated dose, frequency and duration in days, per patient was 22 in the TH and 8 in the NTH (Table 3). The duration of the hospital stay of the patients in the TH was higher than that of the patients in the NTH (mean 10.1 days at the TH and 4.4 days at the NTH, *P* < 0.05).

**Table 2:** Total number of admissions and included patients each year at the two Indian private-sector hospitals

	NTH		TH	
Year	Number of admissions, n	Patients included, n (%)	Number of admissions, n	Patients included, n (%)
From April 1st, 2008	10,480	230 (2)	6965	80 (1)
2009	15,384	247 (2)	10,369	103 (1)
2010	16,126	311 (2)	11,145	118 (1)
2011	15,136	288 (2)	12,188	171 (1)
2012	14,414	264 (2)	10,454	139 (1)
2013	14,627	267 (2)	9821	146 (1)
2014	16,473	268 (2)	13,186	208 (2)
2015	13,740	249 (2)	12,387	124 (1)
2016	14,165	310 (2)	16,741	128 (1)
Until May 31st, 2017	4121	70 (2)	5852	45 (1)
Total	134,666	2504 (1)	109,108	1262 (1)

Notes: Values are presented in total number of admissions, number of included patients and percentage of total number of admissions

Abbreviations: *n* Number; *NTH* Non-teaching hospital, *TH* Teaching hospital

**Table 3:** Clinical characteristics and antibiotic prescribing among patients with severe infections at two private sector hospitals

	NTH, n (%)	TH, n (%)	Odds ratio (95% CI)	***P***-value
**All included patients,** **n (%)**	2504 (100)	1262 (100)		
Mean age	49.2	47.1		**< 0.01**
Women, n (%)	894 (36)	301 (24)		
Men, n (%)	1610 (64)	961 (76)		
Patients prescribed ABs, n (%)	2294 (92)	1122 (89)	**1.36 (1.08, 1.72)**	**< 0.05**
AB prescriptions, n (n of prescriptions per patients prescribed AB)	18,751 (8)	24,956 (22)		
**Cellulitis**	388 (15)	402 (32)		
Mean age	50.4	48.7		0.16
Women, n (%)	101 (26)	79 (20)		
Men, n (%)	287 (74)	323 (80)		
Patients prescribed ABs, n (%)	354 (91)	362 (90)	1.15 (0.69,1.91)	0.57
AB prescriptions, n (n of prescriptions per patients prescribed AB)	3505 (10)	8608 (24)		
**Endocarditis**	7 (0)	2 (0)		
Mean age	45.6	35.0		0.21
Women, n (%)	3 (43)	2 (100)		
Men, n (%)	4 (57)	0 (0)		
Patients prescribed ABs, n (%)	6 (86)	1 (50)	6 (0.04,547.49)	0.28
AB prescriptions, n (n of prescriptions per patients prescribed AB)	33 (6)	54 (54)		
**Epiglottitis**	12 (0)	1 (0)		
Mean age	35.8	40.0		0.25
Women, n (%)	6 (50)	0 (0)		
Men, n (%)	6 (50)	1 (100)		
Patients prescribed ABs, n (%)	11 (92)	1 (100)	–	–
AB prescriptions, n (n of prescriptions per patients prescribed AB)	57 (5)	2 (2)		
**Meningitis**	186 (7)	38 (3)		
Mean age	40.6	37.0		0.24
Women, n (%)	90 (48)	15 (39)		
Men, n (%)	96 (52)	23 (61)		
Patients prescribed ABs, n (%)	167 (90)	27 (71)	**3.58 (1.37, 8.93)**	**< 0.05**
AB prescriptions, n (n of prescriptions per patients prescribed AB)	1126 (7)	442 (16)		
**Peritonitis**	431 (17)	252 (20)		
Mean age	44.4	44.0		0.71
Women, n (%)	52 (12)	35 (14)		
Men, n (%)	379 (88)	217 (86)		
Patients prescribed ABs, n (%)	402 (93)	233 (92)	1.13 (0.58, 2.14)	0.69
AB prescriptions, n (n of prescriptions per patients prescribed AB)	4909 (12)	6715 (29)		
**Pneumonia**	761 (30)	410 (32)		
Mean age	49.6	48.4		0.30
Women, n (%)	294 (39)	100 (24)		
Men, n (%)	467 (61)	310 (76)		
Patients prescribed ABs, n (%)	692 (91)	366 (89)	1.21 (0.78, 1.83)	0.36
AB prescriptions, n (n of prescriptions per patients prescribed AB)	4686 (7)	6179 (17)		
**Pyelonephritis**	71 (3)	3 (0)		
Mean age	42.3	26.7		**< 0.01**
Women, n (%)	32 (45)	2 (67)		
Men, n (%)	39 (55)	1 (33)		
Patients prescribed ABs, n (%)	68 (96)	2 (67)	11.33 (0.14,262.11)	0.16
AB prescriptions, n (n of prescriptions per patients prescribed AB)	518 (8)	89 (45)		
**Septic arthritis**	3 (0)	38 (3)		
Mean age	43.3	45.0		0.93
Women, n (%)	1 (33)	15 (39)		
Men, n (%)	2 (67)	23 (61)		
Patients prescribed ABs, n (%)	3 (100)	29 (76)	–	–
AB prescriptions, n (n of prescriptions per patients prescribed AB)	15 (5)	1159 (40)		
**Sepsis**	645 (26)	116 (9)		
Mean age	54.9	49.2		**< 0.01**
Women, n (%)	315 (49)	53 (46)		
Men, n (%)	330 (51)	63 (54)		
Patients prescribed ABs, n (%)	591 (92)	101 (87)	1.6 (0.82,3.0)	0.11
AB prescriptions, n (n of prescriptions per patients prescribed AB)	3902 (7)	1708 (17)		

Notes: *P*-values for mean age were obtained by linear regression. Odds ratios, p-values and CIs for antibiotic prescribing were obtained by chi-square tests. Statistically significant *p*-values are marked in bold font

*Abbreviations*: *AB* Antibiotic, *CI* Confidence interval, *n* Number, *NTH* Non-teaching hospital, *OR* Odds ratio, *TH* Teaching hospital

**Fig. 2:**
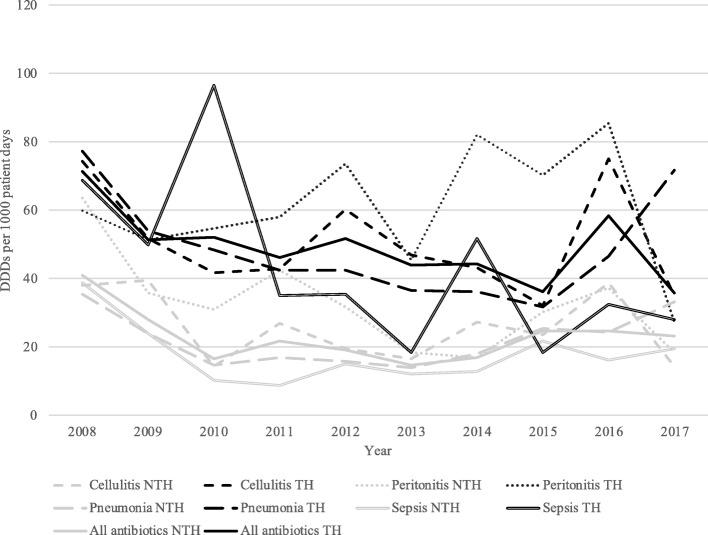
Prescribing of antibiotics among patients with severe infections from 2008 to 2017. Prescribing is quantified and presented in DDDs per 1000 patients (y-axis) each year (x-axis) for selected infectious diseases for each hospital. Trends for each slope, indicating an overall postitive or negative trend of antibiotic prescribing (measured in DDDs per 1000 patients), over the study period are obtained by linear regression analysis and are presented together with *P*-values in Table 4.Abbreviations: DDD, defined daily dosis; NTH, non-teaching hospital; TH, teaching hospital

**Fig. 3:**
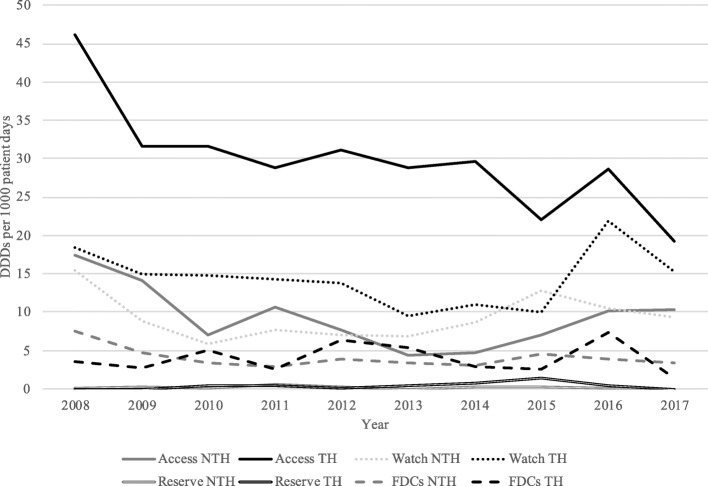
Prescribing of antibiotics categorized by *access, watch, reserve* and FDCs of antibiotics from 2008 to 2017. Prescribing is quantified and presented in DDDs per 1000 patients (y-axis) each year (x-axis) of the total prescribing of each antibiotic cathegory (access, watch, reserve and FDCs of antibiotics) for each hospital. Trends for each slope, indicating an overall postitive or negative trend of antibiotic prescribing (measured in DDDs per 1000 patients), over the study period are obtained by linear regression analysis and are presented together with *P*-values in Table 4. Abbreviations: DDD, defined daily dosis; FDC, fixed dose combinations; NTH, non-teaching hospital; TH, teaching hospital

Prescribing of antibiotics for all indications increased between 2008 and 2017 in the NTH (*P* < 0.01), whereas prescribing practices did not change significantly during this period in the TH (*P* = 0.07, Fig. 2, Table 4). Antibiotics included in the *access* category comprised 61% of the total antibiotics prescribed in the TH and 40% of the total prescribed in the NTH (*P* < 0.01, Fig. 3, Table 4). Prescribing of *access* antibiotics increased in the NTH between 2008 and 2017. Prescribing of antibiotics categorized as *watch* antibiotics comprised 29% of the total antibiotics prescribed in the TH and 40% of the total prescribed in the NTH. Prescribing of *watch* antibiotics rose in both hospitals between 2008 and 2017 (*P* < 0.01 for both hospitals, Fig. 3, Table 4). *Reserve* antibiotics comprised less than 1 % antibiotics prescribed in both hospitals. However, prescribing of *reserve* antibiotics increased between 2008 and 2017 in the TH (*P* < 0.01, Fig. 3, Table 4).

**Table 4:** Description of trends in antibiotic prescribing among patients with severe infections in Ujjain between 2008 and 2017

	NTH	TH
**Antibiotic prescribing among specific diagnoses**		
All antibiotics	**13.84 (< 0.01)**	1.82 (0.07)
Cellulitis	**5.72 (< 0.01)**	**6.52 (< 0.01)**
Peritonitis	**14.59 (< 0.01)**	**18.52 (< 0.01)**
Pneumonia	**4.87 (< 0.01)**	**7.30 (< 0.01)**
Sepsis	**2.18 (0.03)**	**−21.91 (< 0.01)**
**Antibiotic prescribing among all included patients**		
*Access* antibiotics	**11.52 (< 0.01)**	1.78 (< 0.07)
*Watch* antibiotics	**9.63 (< 0.01)**	**6.49 (< 0.01)**
*Reserve* antibiotics	−0.76 (0.45)	**2.54 (< 0.01)**
FDCs of antibiotics	**14.28 (< 0.01)**	**7.31 (< 0.01)**
**Antibiotic prescribing among sepsis patients**		
*Access* antibiotics	1.49 (0.14)	**−16.89 (< 0.01)**
*Watch* antibiotics	**3.02 (< 0.01)**	**−11.38 (< 0.01)**
*Reserve* antibiotics	**−9.32 (< 0.01)**	Too few prescriptions
FDCs of antibiotics	**3.78 (< 0.01)**	**−9.93 (< 0.01)**

Notes: All values are presented with a value for the slope: t, followed by *P*-value in parenthesis. The t-value is obtained from linear regression analysis and indicates a postitive or negative trend of antibiotic prescribing (measured in DDDs per 1000 patients), over the study period. A positive t-value shows a positive trend of antibiotic prescribing during the study period and a negative t-value shows a negative trend of antibiotic prescribing during the study period. Statistically significant *p*-values indicates a significant trend and are marked in bold font

*Abbreviations*: *DDD* Defined daily dosis, *FDC* Fixed dose combination, *NTH* Non-teaching hospital, *TH* Teaching hospital

The overall antibiotic prescribing among all included patients increased from 2008 to 2017 at the NTH (*P* < 0.01) but did not significantly change at the TH (*P* = 0.07, Fig. 1, Table 4). Antibiotics included in the *access* category comprised 61% of the total antibiotic prescribing at the TH and 40% at the NTH. Prescribing of *access* antibiotics increased at the NTH from 2008 to 2017 (*P* < 0.01, Fig. 3, Table 4). Prescribing of antibiotics categorized as *watch* antibiotics comprised 29% of the total antibiotic prescribing at the TH and 40% at the NTH. Prescribing of *watch* antibiotics increased at both hospitals from 2008 to 2017 (*P* < 0.01 for both hospitals, Fig. 3, Table 4). *Reserve* antibiotics comprised less than 1 % of the antibiotic prescribing at both hospitals, however prescribing of *reserve* antibiotics increased from 2008 to 2017 at the TH (*P* < 0.01, Fig. 3, Table 4). Prescribing of FDCs of antibiotics (J01R) comprised 8% of the antibiotic prescribing at the TH and 18% at the NTH. Prescribing of FDCs increased at both hospitals from 2008 to 2017 (*P* < 0.01 for both hospitals, Fig. 3, Table 4).

Patients diagnosed of cellulitis, peritonitis, pneumonia and sepsis accounted for 88% of the admissions to the NTH and 93% of the admissions in the TH. Table 5 shows the antibiotic groups (first-choice or second-choice) prescribed for each of these four diagnoses, which accounted for 75% of all antibiotics prescribed. Total antibiotic prescribing among patients with cellulitis, peritonitis and pneumonia increased between 2008 and 2017 in both the hospitals (*P* < 0.01 for both hospitals, Fig. 2, Table 4). Antibiotic prescribing among patients with sepsis increased between 2008 and 2017 in the NTH (*P* = 0.03) but decreased in the TH (*P* < 0.01, Fig. 2, Table 4). Sepsis was the only one of the four diagnoses where antibiotic consumption decreased at one of the hospitals.

**Table 5:** Antibiotics prescribed among patients with severe infections at two private sector hospitals from 2007 to 2018

	Antibiotic prescribing in DDDs per 1000 patient days										%	Slope, t^**a**^	***P***-value
Year	2008	2009	2010	2011	2012	2013	2014	2015	2016	2017			
**Cellulitis NTH**													
**J01CR**	4.12	4.44	0.73	4.78	3.17	4.90	5.77	4.33	3.24	7.11	16	2.32	**0.02**
**J01D**	13.11	9.01	1.98	11.52	5.09	4.28	12.50	7.54	14.18	3.06	32	6.33	**< 0.01**
**J01R**	6.53	5.60	3.12	2.60	4.70	3.46	3.01	6.78	6.12	1.84	17	6.97	**< 0.01**
**J01X**	9.25	15.76	3.68	3.34	1.30	1.05	1.62	1.08	1.42	1.48	15	3.31	**< 0.01**
**Cellulitis TH**													
**J01CR**	10.84	7.45	4.24	7.14	9.47	8.99	10.07	8.03	16.29	13.33	19	4.91	**< 0.01**
**J01D**	4.48	6.01	9.32	6.22	4.76	4.66	6.87	6.53	9.98	2.10	12	5.58	**< 0.01**
**J01G**	9.33	13.53	9.11	9.26	14.55	10.47	10.01	6.37	15.64	5.45	21	0.44	0.66
**J01M**	10.20	8.70	3.35	5.66	9.00	4.51	4.30	3.31	9.11	6.22	13	2.66	**< 0.01**
**J01X**	6.53	3.70	7.38	6.50	6.86	10.22	7.89	3.84	9.17	3.91	13	2.27	**0.02**
**Peritonitis NTH**													
**J01CR**	3.71	4.26	3.85	10.23	3.06	5.70	4.45	5.89	5.67	4.40	16	4.98	**< 0.01**
**J01D**	26.28	6.43	4.54	4.34	10.36	7.35	1.41	7.78	8.04	5.06	25	1.34	0.18
**J01R**	8.58	6.14	5.29	5.55	5.01	1.14	3.09	5.02	6.19	1.40	15	8.67	**< 0.01**
**J01X**	16.03	11.72	11.64	9.09	4.57	2.49	1.58	1.81	3.54	1.97	20	10.95	**< 0.01**
**Peritonitis TH**													
**J01CR**	5.84	4.39	6.68	5.55	10.92	9.13	16.54	13.44	13.36	5.35	15	10.16	**< 0.01**
**J01D**	4.00	4.95	9.63	9.44	6.87	2.76	6.04	7.64	15.87	7.73	12	17.55	**< 0.01**
**J01G**	10.08	4.65	11.08	9.94	11.84	6.49	11.43	11.87	8.64	4.08	15	8.13	**< 0.01**
**J01M**	16.05	10.72	3.27	6.40	11.99	5.83	11.60	12.86	15.42	3.56	16	2.92	**< 0.01**
**J01X**	15.75	9.55	16.85	17.73	15.44	13.92	22.64	17.30	23.05	5.24	26	8.69	**< 0.01**
**Pneumonia NTH**													
**J01CR**	11.54	5.01	4.05	4.27	4.95	4.61	5.96	5.79	8.79	8.17	29	−0.91	0.36
**J01D**	7.75	6.92	4.22	8.53	3.24	2.95	1.37	4.44	4.23	4.18	22	−1.38	0.17
**J01M**	1.00	0.49	0.47	0.50	2.41	0.83	3.98	7.34	3.47	1.18	10	2.54	**0.01**
**J01R**	5.74	4.61	3.51	2.55	4.21	4.76	5.27	4.61	3.72	4.82	20	5.65	**< 0.01**
**Pneumonia TH**													
**J01A**	16.44	11.85	14.23	8.45	13.76	10.18	7.21	2.91	2.25	18.79	25	1.81	0.07
**J01CR**	8.45	12.64	10.66	1.91	8.26	15.71	20.02	19.21	15.38	13.27	30	6.65	**< 0.01**
**J01D**	3.85	6.01	5.04	10.91	7.24	4.65	3.94	3.81	15.31	20.74	19	12.87	**< 0.01**
**J01M**	15.87	9.36	8.72	5.72	3.88	2.04	1.61	1.77	4.65	7.44	14	1.89	0.06
**Sepsis NTH**													
**J01CR**	5.05	3.90	2.13	1.97	2.23	2.55	3.01	6.90	3.47	6.66	21	1.10	0.27
**J01D**	10.74	7.47	1.19	3.27	4.63	3.86	4.77	6.37	4.29	1.70	27	−4.67	**< 0.01**
**J01R**	7.55	3.66	1.71	1.33	2.09	3.36	1.84	3.07	2.63	2.76	17	3.17	**< 0.01**
**J01X**	12.22	6.54	4.51	0.99	0.79	0.00	1.28	1.02	0.72	0.00	16	−6.07	**< 0.01**
**Sepsis TH**													
**J01A**	14.61	7.67	9.59	1.83	4.31	2.95	4.26	0.00	0.00	3.13	11	−5.50	**< 0.01**
**J01CR**	1.84	11.18	27.87	2.76	5.09	1.83	8.04	7.09	7.79	7.09	19	−5.07	**< 0.01**
**J01D**	1.37	5.48	8.48	3.03	5.87	3.79	12.27	5.57	7.21	4.89	13	−5.20	**< 0.01**
**J01M**	11.51	1.75	14.45	11.32	3.13	0.11	4.75	0.91	2.81	3.62	13	−8.49	**< 0.01**
**J01X**	8.95	12.93	15.34	7.08	9.18	6.36	12.60	0.91	6.00	4.07	19	−15.48	**< 0.01**

Notes: Antibiotics presented comprise ≥ 75% of the total antibiotic prescribing within each diagnosis group. Numbers are presented in total antibiotic prescribing of antibiotic groups for each year, measured in DDD/1000 patient days and percentage of the total prescribing of antibiotics for each diagnosis and hospitals during the study period. ^a^ t is obtained by linear regression, a positive t shows a positive trend and a negative t shows a negative trend of prescribing. Statistically significant p-values indicates a significant trend and are marked in bold font

*Abbreviations*: *DDD* Defined daily doses, *NTH* Non-teaching hospital, *TH* Teaching hospital

### Cellulitis

In the NTH, the two most commonly prescribed antibiotics were second-choice treatments: J01D (32% of total antibiotics prescribed) and J01R (17%) [15]. Prescribing of both J01D and J01R increased during the study period, as did prescribing of the recommended treatments (J01CR and J01D) (*P* < 0.05 for all, Table 5). In the TH, the most commonly prescribed antibiotics were from the J01CR (19%) and J01G group (21%) and prescribing of J01CR increased between 2008 and 2017. (*P* < 0.01, Table 5). At the NTH, prescribing of *access, watch* and FDCs (J01R) increased from 2008 to 2017 while at the TH, prescribing of *access, watch, reserve* and FDCs increased (*P* < 0.01 for all categories, at both hospitals).

### Peritonitis

In the NTH, the two most commonly prescribed antibiotics were first-choice treatments for community acquired peritonitis [18]: J01D antibiotics comprised 25% of antibiotics prescribed, and J01X accounted for 20% of antibiotics prescribed. Prescribing of J01X increased during the study period (*P* < 0.01, Table 5). In the TH, the two most commonly prescribed antibiotics were first-choice treatments: J01M (16%) and J01X (26%), and prescribing of both J01M and J01X groups increased between 2008 and 2017 (*P* < 0.01 for both antibiotic groups, Table 5). In addition, prescribing of J01CR (first-choice treatment for community-acquired peritonitis) increased in both hospitals during the study period. At both hospitals, prescribing of *access, watch, reserve* and FDCs (J01R) increased from 2008 to 2017 (*P* < 0.01 for all categories mentioned at both hospitals).

### Pneumonia

In the NTH, the beta-lactam antibiotics (J01D) and combinations of penicillins (J01CR) were two most commonly prescribed. J01D antibiotics are listed as first-choice treatment for community-acquired pneumonia, and J01CR antibiotics are listed as second-choice treatment for community-acquired and as first-choice treatment for health-care acquired pneumonia (Table 5) [15]. J01CR and J01D antibiotics comprised 29 and 22%, respectively, of prescribed antibiotics in the diagnosis group. Overall, the prescribing practices did not change between 2008 and 2017. In the TH, the two most commonly prescribed antibiotics were from the J01A and J01CR groups. J01A group is listed as second-choice treatment for community acquired pneumonia, and J01CR is listed as second-choice treatment for community and as first-choice treatment for health-care acquired pneumonia [15]. The prescribing of J01CR increased from 2008 to 2017 (*P* < 0.01, Table 5). J01CR and J01D antibiotics comprised 25 and 30%, respectively, of antibiotics prescribed for pneumonia in the TH. Prescribing of FDCs (J01R) declined (*P* < 0.01) for all categories in both hospitals. In the NTH, prescribing of *access, watch* and FDC antibiotics increased between 2008 and 2017 and in the TH, prescribing of *access* and *watch* antibiotics rose between 2008 and 2017.

### Sepsis

In both hospitals, piperacillin with tazobactam (J01CR) group was commonly prescribed, which is adherent with guidelines [19]. In the NTH, J01CR antibiotics accounted for 21% of all antibiotics prescribed, and J01D accounted for 27% of all antibiotics prescribed. Prescribing of J01D decreased between 2008 and 2017 (*P* < 0.01, Table 5). In the TH, the most commonly prescribed antibiotics were from the J01CR (19%) and J01X groups (19%) and prescribing of both J01CR and J01X decreased between 2008 and 2017. (*P* < 0.01 for both antibiotic groups, Table 5). At the NTH, prescribing of *watch* and FDCs (J01R) increased from 2008 to 2017 while prescribing of *reserve* antibiotics decreased. At the TH, prescribing in DDDs per 1000 patient days, of *access*, *watch* and FDCs decreased from 2008 to 2017 (Table 4).

## Discussion

According to our knowledge, this is the first study that compares antibiotic prescribing practices for selected infectious diagnoses over a 10 years period in two Indian private-sector hospitals. In the NTH, prescribing of antibiotics for all indications, including antibiotics specifically for cellulitis, pneumonia, peritonitis and sepsis diagnoses, increased from 2008 to 2017. In the TH, although antibiotic prescribing practices did not change during the study period, antibiotic prescribing for cellulitis, pneumonia and peritonitis increased and decreased for sepsis. Between 2008 and 2017, prescribing of *access*, *watch* and FDC of antibiotics rose in the NTH, and prescribing of *watch*, *reserve* and FDCs increased in the TH.

In this study, we analysed adherence to various international recommendations for antibiotic prescribing: the WHO’s recommendations for empirical antibiotic treatment for cellulitis and pneumonia, the recommendations for empirical antibiotic treatment from the ‘Surviving sepsis campaign’ for sepsis and the recommendations of the World Society of Emergency Surgery for peritonitis [15, 18, 19]. Adherence to these international guidelines increased in the TH compared to the NTH during the study period, especially prescribing of antibiotics for peritonitis and pneumonia.

Guidelines on empirical antibiotic treatment are often based on whether an infection is healthcare associated or community acquired. As both bacterial flora and susceptibility patterns vary worldwide, the following factors are important to consider while selecting the most appropriate antibiotic: the bacterium most likely to be the cause of the infection, patient’s clinical status, allergies to specific antibiotics and current or previous antibiotic resistance and responses to antibiotic treatment [1, 18, 19]. In this study, most of the patients did not have cultures sent for analysis, as the use of microbiological analyses was limited at both hospitals. The lack of microbiological analyses makes it difficult to comment on the rationale underlying the antibiotic prescription practices in the hospitals. However, prescribing of broad-spectrum antibiotics in both hospitals was high, which is in line with previous reports on prescribing of broad-spectrum antibiotics in Indian hospitals [12, 20]. As all the patients in the NTH paid for the treatment they received, the patients might have put pressure on their physicians to prescribe broad-spectrum antibiotics. In a qualitative study including 36 Indian doctors, Kotwani et al. reported that doctors faced demands from their patients to prescribe ‘strong’ antibiotics and that they sometimes prescribed antibiotics because they did not have time to debate with patients due to time constraints in busy health care facilities [21]. The aforementioned factors, as well as the desire to avoid re-consultation, might have contributed to the prescribing of broad-spectrum antibiotics in the hospitals in the present study.

In the NTH, the FDCs of antibiotics (J01R) were commonly prescribed to all patients, including those with cellulitis, peritonitis, pneumonia and sepsis. In contrast, the FDCs were less frequently prescribed in the TH. Prescribing of the FDCs for all indications increased in the TH during the study period but decreased for diagnoses of pneumonia and sepsis. Prescribing FDCs of antibiotics is not recommended, as they have been shown to drive antibiotic resistance, a common consequence of unnecessarily prescribed antibiotics, often in incorrect doses [22]. Appropriate prescribing of antibiotics requires that the dose be tailored for the individual patient, which is often not possible while prescribing the FDCs. A few FDCs that includes unapproved formulations, are known to be widely used in India [23–26]. In March 2016, the Indian Government banned around 330 FDCs of drugs, of which 63 (19%) were FDCs of antibiotics [27]. However, there are still more than 118 FDCs of antibiotics available in the Indian market [27]. The presence of medical representatives and lack of local prescribing guidelines may have contributed to the higher prescribing of FDCs of antibiotics in the NTH as compared with that in the TH, where medical representatives are forbidden, and mainly generic medicines are procured by the management. Previous research demonstrated that pressure from pharmaceutical companies influence physicians’ prescribing practices in India [28].

Regardless of the country or setting, *access* antibiotics should primarily be used to save, whereas *watch* and *reserve* antibiotics should be used only for specific and limited indications in critically ill patients or patients with infections caused by bacteria with known antibiotic resistance [17]. In both hospitals, *reserve* antibiotics comprised less than 1% of the total antibiotics prescribed. In the TH, *access* antibiotics were most commonly prescribed (61% of antibiotics prescribed), followed by *watch* antibiotics (29%). However, in the NTH, *access* and *watch* antibiotics were prescribed in equal numbers (40% each). These results indicated that *watch* antibiotics accounted for a higher proportion of antibiotics prescribed in the NTH than in the TH. Furthermore, in the TH, prescribing of *watch* and *reserve* antibiotics for cellulitis or peritonitis increased in both hospitals during the study period, whereas prescribing of these antibiotics for sepsis decreased.

As noted earlier, the lack of microbiological analyses makes it difficult to comment on the rationale underlying the antibiotic prescription practices in the two hospitals. Nevertheless, the prescribing practices can be considered in terms of international recommendations on antibiotic aimed at reducing the emergence of antibiotic resistance. Based on the results of the present study, greater adherence to international guidelines for empirical antibiotic treatment is needed at both hospitals to optimize antibiotic use. The *access, watch* and *reserve* categorization of antibiotics provides a practical guide for proper antibiotic prescribing and can provide a basis for the development of local prescribing guidelines [17].

The relatively low incidence of some infectious diseases, such as infective endocarditis, among admissions to the NTH and the TH may be explained by underdiagnosing, which is a major problem in hospitals, as described in a previous study on infective endocarditis in India [29]. In many health care facilities in LMICs, microbiological tests and imaging methods are seldom used due to a lack of access to these diagnostic methods or a lack of time and money [8, 21]. In only a small number of cases in the present study, samples were sent for microbiological analyses, despite such analyses being readily available in both hospitals. Patient- and prescriber-related factors have been put forward to explain why culture tests are not routinely performed in the hospitals [12, 21, 30]. Patient-related factors include patients not being able to afford the tests or prefer to stay for short periods to pay less at the NTH. Prescriber-related factors might include doctors not having the time to wait for lab results due to overcrowding in the hospitals or an additional factor may be monetary driven, that doctors are paid for the number of patients they admit to the hospital so they might be wishing to see as many patients as possible in a given period [12, 21, 30]. Routine use of diagnostic methods, such as microbiological analysis and imaging methods, for patients with suspected infections might contribute to better management of and guidance on antibiotic treatment for infectious diseases to reduce antibiotic overuse.

### Methodological considerations

#### Strengths and limitations

A strength of this study is the data collection design. The hospitals included in this study lacked computerized medial record systems, and the data were collected manually, using the same method over a long period of time. As data collection and data entry in the registry were performed manually, there was a risk of missing data. To minimize this risk, the staff who completed the forms and data entry were trained at regular intervals. Another strength of this study was that the same form was used for data collection at both hospitals, which enabled comparisons of antibiotic prescribing between the hospitals. A limitation of this study was the absence of medical records and documentation of previous medical history from the included patients. Since there were no medical records or documentation available, no predisposing factors among the patients could be evaluated. Another limitation was that none of the hospitals used microbiological analysis (cultures) consistently. Consequently, most of the diagnoses were based on clinical suspicion. As almost all antibiotic prescribing was empirical, it was not possible to assess whether the antibiotics were rationally prescribed. However, by applying the WHO’s antibiotic categories of *access, watch* and *reserve,* as well as existing guidelines on empirical prescribing for each diagnosis, the appropriateness of antibiotic prescribing practices in both hospitals could be assessed. Finally, this study included only adult patients. The reasons for this were two-fold: First, the ‘defined daily dose’ system is based on adult patients. Thus, antibiotic use among paediatric patients cannot be evaluated using the defined daily dose system. Second, the recommendations used to assess rationality in antibiotic prescribing in this study were for adults only.

## Conclusions

Over the 10 years period, prescribing of all antibiotic categories, including *access, watch* and FDCs of antibiotics, increased in the NTH. In the TH, antibiotic prescribing practices did not change significantly, although prescribing of *watch, reserve* and FDCs of antibiotics increased between 2008 and 2017. Antibiotic prescribing for pneumonia, peritonitis and cellulitis increased in both hospitals between 2008 and 2017. In the TH, antibiotic prescribing for sepsis decreased but increased in the NTH. The results indicate that antibiotic prescribing practices need to be improved in both hospitals, although the TH generally prescribed more recommended antibiotics and fewer FDCs. Furthermore, the prescribing of recommended antibiotics improved over the study period in the TH. Factors contributing to extensive prescribing of *watch* antibiotics and FDCs in the NTH could be pressure from pharmaceutical companies and patients, as well as a lack of a local prescribing guidelines. The establishment of antibiotic stewardship programs based on the *access, watch* and *reserve* antibiotic categorization, as well as the implementation of locally adapted lists of essential medicines and prescribing guidelines could contribute to improved antibiotic prescribing practices and thus limit the development of antibiotic resistance. Furthermore, the implementation of routine diagnostic methods, such as microbiological analysis, could improve the management of infectious diseases and guide antibiotic therapy decisions for appropriate prescribing of antibiotics. The hospitals included in the present study are typical of those in similar settings in LMICs, and our results are in line with those of previously published studies in this area. The results highlight areas for improvement in antibiotic prescribing practices and same could be anticipated from other similar settings.

## Data Availability

The data that support the findings of this study, contains patients’ identifying information thus restrictions apply to the availability of these data to make it publicly available, as per the institutional policy. Data, however, can be made available upon reasonable request and with permission of the Institutional ethics committee; through the Chairman, Ethics Committee of Ruxmaniben Deepchand Gardi Medical College in Ujjain, India, 456006.

## Abbreviations

ATC: Anatomical Therapeutic Chemical classification system
CI: Confidence interval
DDD: Defined Daily Doses
ESBL: Extended spectrum beta lactamase
GLASS: Global Antimicrobial Surveillance System
LMIC: Low- and middle-income countries
NTH: Non-teaching hospital
TH: Teaching hospital
VRE: Vancomycin-resistant enterococci
WHO: World Health Organization
